# Supplementary material for: Studying Corrosion Using Miniaturized Particle Attached Working Electrodes and the Nafion Membrane
Source: Micromachines (Basel). 2021 Nov 18;12(11):1414. doi: 10.3390/mi12111414 (PMC8618753; doi:10.3390/mi12111414)
Supplement: Supplementary file 1 [file micromachines-12-01414-s001.zip › micromachines-1452243-SI.pdf]

micromachines

MDPI

Supporting Information

Studying Corrosion Using Miniaturized Particle Attached Working Electrodes and the Nafion Membrane

Jiyoung Son [1], Edgar C. Buck [1], Shawn L. Riechers [1], Shalini Tripathi [1], Lyndi E. Strange [1], Mark H. Engelhard [2] and Xiao-Ying Yu [1,*]

[1] Energy and Environment Directorate, Pacific Northwest National Laboratory, Richland, WA 99354; jiyoung.son@pnnl.gov (J.S.); edgar.buck@pnnl.gov (E.C.B.); shawn.riechers@pnnl.gov (S.L.R.); shalini.tripathi@pnnl.gov (S.T.); lyndi.strange@pnnl.gov (L.E.S.)

[2] Environmental Molecular Sciences Laboratory, Pacific Northwest National Laboratory, Richland, WA 99354; mark.engelhard@pnnl.gov

* Correspondence: xiaoying.yu@pnnl.gov

Table of Contents

Supporting Experimental Details S-3
Fabrication of SALVI E-cell device S-3
Static ToF-SIMS conditions S-3
Additional XPS fitting conditions S-3
Supporting Figures S-4
Figure S1. (a) The optical microscopy image of the as-made CeO_2 Nafion working electrode (WE) and (b) The profilometer measurement image of the CeO_2 Nafion WE. S-4
Figure S2. The CeO_2 WE CV plots with different Nafion fabrication conditions: (a) CeO_2 WE 5 wt% Nafion with 1000 rpm spin rate, (b) CeO_2 WE 5 wt% Nafion with 500 rpm spin rate, (c) CeO_2 WE 5 wt% Nafion with drop application, (d) CeO_2 WE 20 wt% Nafion with 1000 rpm spin rate, (e) CeO_2 WE 20 wt% Nafion with 500 rpm spin rate, and (f) CeO_2 WE 20 wt% Nafion with drop application. The scanning rate is at 100 mV/s. S-6
Figure S3. Reproducibility of optimized CeO_2 devices using (a) 100 mV/s scan rate, (b) 80 mV/s scan rate, and (c) The current vs. square root of scan rate plot. Ipa refers to oxidation peak current and Ipc reduction peak current. S-6
Figure S4. SEM FIB images of (a) the trench of the CeO_2 WE after electrochemical corrosion and (b) the pristine CeO_2 WE, using 6500 times magnification. S-7
Figure S5. ToF-SIMS spectral results in (a) the corroded CeO_2 WE after electrochemical corrosion and (b) the pristine CeO_2 WE in the mass range of m/z^+ 1–355. .... S-8
Figure S6. ToF-SIMS spectral results of (a) the corroded CeO_2 WE after electrochemical corrosion and (b) the pristine CeO_2 WE in the mass range of m/z^+ 1–355. .... S-9
Figure S7. XPS wide scan spectral results of (a) the corroded CeO_2 WE after electrochemical corrosion, (b) the pristine CeO_2 WE, and (c) the Nafion membrane. S-10
Figure S8. Normalized Ce(3d) spectra before and after cyclic voltammetry. Regions indicative of Ce^{3+} are marked (1), (2), and (3), respectively. S-11
Supplemental Tables S-12
Table S1. XPS peak positions and corresponding FWHM values for the electrode analyzed before CV. S-12
Table S2. XPS peak positions and corresponding FWHM values for the electrode analyzed after CV. S-13
References S-14

Supporting Experimental Details

Fabrication of SALVI E-cell device

Fabrication of *SALVI E-cell* is based on previous works [1,2]. In brief, the fabricated WE will be bond together with an electrochemical cell chamber made of polydimethylsiloxane (PDMS) block by plasma surface activation. An electrochemical chamber is ellipsoid shape width 7 mm long, 3 mm wide, and 1.5 mm depth. Four of holes were punched through the PDMS at each end of the microchannel for fluid injection tubing and CE/RE using a biopsy punch (WPI, Sarasota, FL, USA). Fluid tubing was made from 90° bended metal tubing to connect the microchannel with the PTFE tubes (Sigma, Aldrich, St. Louis, MI, USA). A piece of silver wire (0.25 mm thick, 5 cm long) (Alfa Aesar, Ward Hill, MA, USA) is attached on Au substrate of WE using the silver epoxy (CircuitWorks, Chemtronics, Kennesaw, GA, USA). Two platinum wires (0.25 mm thick, 5 cm long) (Alfa Aesar, Ward Hill, MA, USA) are inserted into the punched hole of PDMS cell chamber. The ends of these two PTFE tubes were connected by a union and fittings (Upchurch, Oak Harbor, WA, USA).

Static ToF-SIMS conditions

The static time-of-flight secondary ion mass spectrometry (ToF-SIMS) analysis were performed using a TOF-SIMS 5 instrument (IONTOF GmbH, Münster, Germany) for mass spectra and 2D mapping measurements of working electrode surfaces. The optimized instrumental conditions were used to obtain the strong molecular ion signals. A pulsed 25 KeV Bi_3^+ ion beam with ~1 pA current and 100 μs cycle time was used as a primary ion source. The secondary ions were emitted at an energy range of 0 to 10 eV [3]. A time-of-flight mass analyzer was used to collect and separate the secondary ions and also to map the surface distributions. The cycle time and current of the beam was kept at 30 μs and ~1200 pA, respectively. The raster resolution was set as 128 × 128 pixels and the raster size was 500 × 500 μm^2. The spectra data scan cycle was set at 60. Initially, the loaded sample set is kept in the loadlock chamber to obtain 3.0×10^{-7} mbar pressure and then all the measurements were carried out at 7×10^{-8} mbar in the main chamber.

Additional XPS fitting conditions

The region between 893 eV and 874 eV was used due to uniform background subtraction for a single region. The discontinuity observed for a single background fit over the entire region is likely due to low concentration of Ce yielding a low signal-to-noise ratio. Furthermore, additional peaks are fixed due to spin orbit coupling (18.10 eV) and peak ratios (3:4). Therefore, fitting the doublet peaks is redundant for quantification purposes.

Supporting Figures

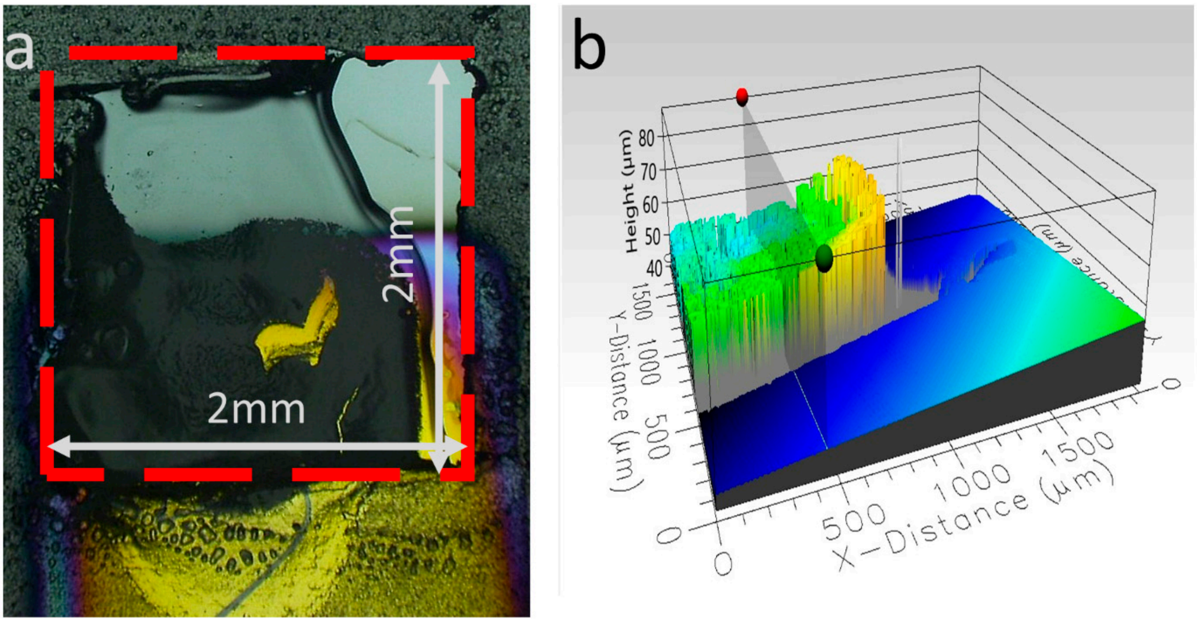

Figure S1. (**a**) The optical microscopy image of the as-made CeO_2 Nafion working electrode (WE) and (**b**) The profilometer measurement image of the CeO_2 Nafion WE.

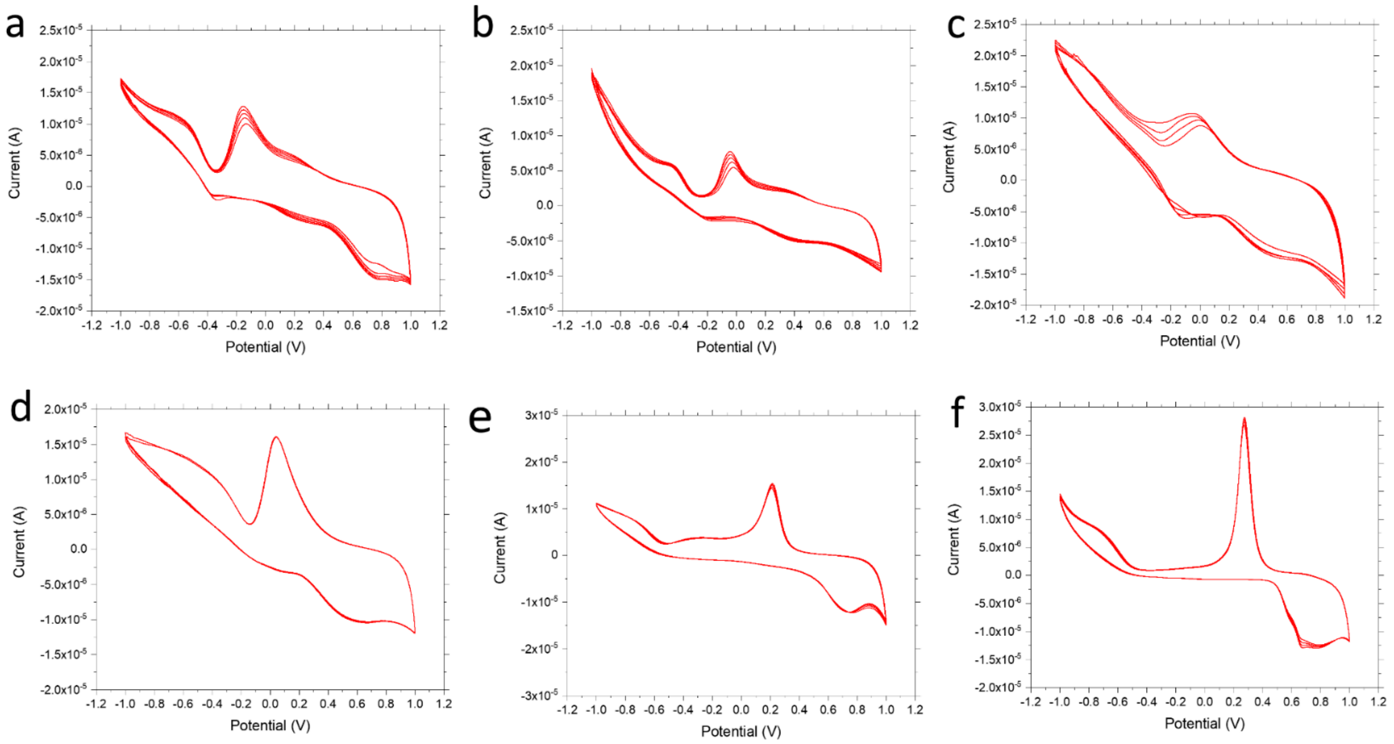

Figure S2. The CeO_2 WE CV plots with different Nafion fabrication conditions: (**a**) CeO_2 WE 5 wt% Nafion with 1000 rpm spin rate, (**b**) CeO_2 WE 5 wt% Nafion with 500 rpm spin rate, (**c**) CeO_2 WE 5 wt% Nafion with drop application, (**d**) CeO_2 WE 20 wt% Nafion with 1000 rpm spin rate, (**e**) CeO_2 WE 20 wt% Nafion with 500 rpm spin rate, and (**f**) CeO_2 WE 20 wt% Nafion with drop application. The scanning rate is at 100 mV/s.

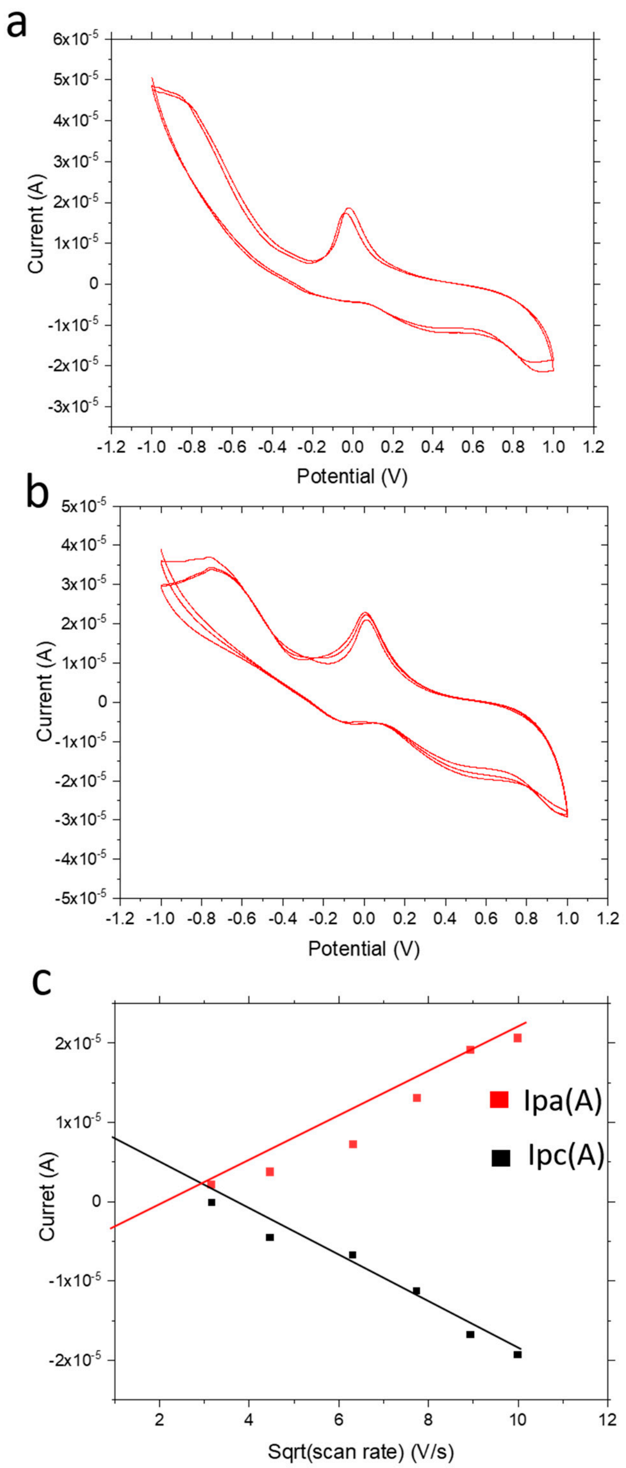

Figure S3. Reproducibility of optimized CeO_2 devices using (**a**) 100 mV/s scan rate, (**b**) 80 mV/s scan rate, and (**c**) The current vs. square root of scan rate plot. Ipa refers to oxidation peak current and Ipc reduction peak current.

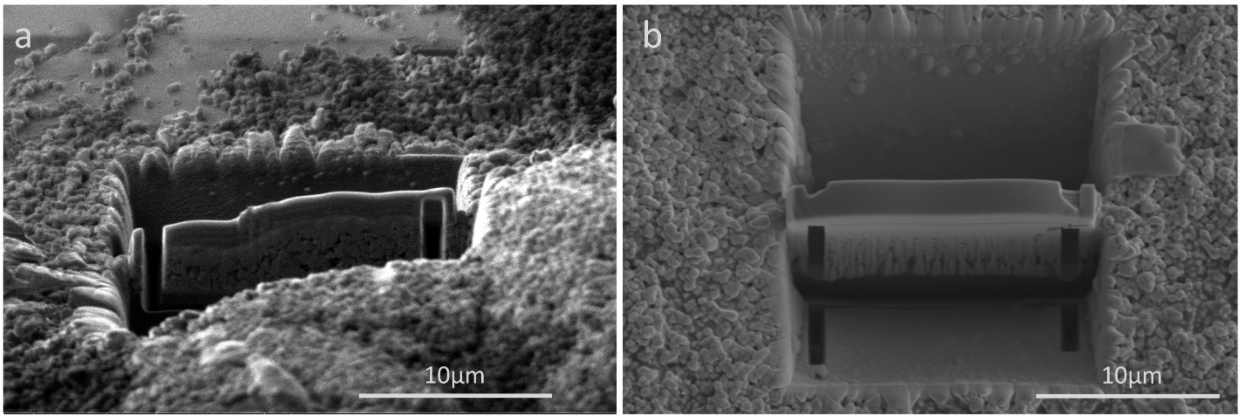

Figure S4. SEM FIB images of (**a**) the trench of the CeO_2 WE after electrochemical corrosion and (**b**) the pristine CeO_2 WE, using 6500 times magnification.

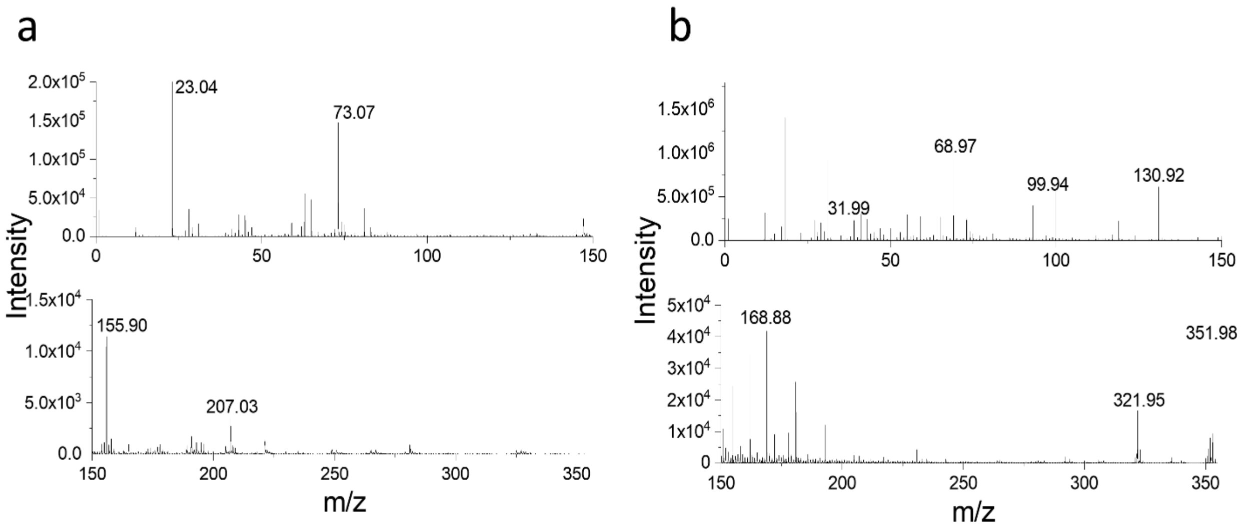

Figure S5. ToF-SIMS spectral results in (**a**) the corroded CeO_2 WE after electrochemical corrosion and (**b**) the pristine CeO_2 WE in the mass range of m/z^+ 1–355.

ToF-SIMS spectral results in **Figure S5** show no signals of sulfur containing fragments in the pristine and corroded CeO_2 WE. Therefore, the speculated sulfur contamination is not a concern using this new approach. Known sulfur peaks are the following, S^+ m/z^+, 31.97, S_2^+ m/z^+ 63.94, S_3^+ m/z^+ 95.91, S_4^+ m/z^+ 127.88, S_5^+ m/z^+ 159.86, S_6^+ m/z^+ 191.83, S_7^+ m/z^+ 223.80, S_8^+ m/z^+ 255.77, S_9^+ m/z^+ 287.74, S_{10}^+ m/z^+ 319.72, and S_{11}^+ m/z^+ 351.69 [4].

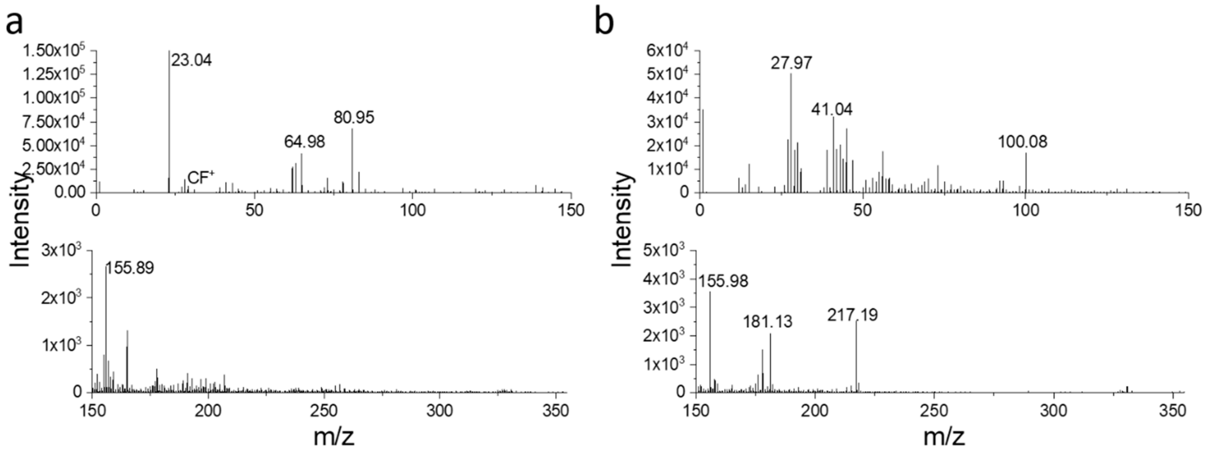

Figure S6. ToF-SIMS spectral results of (**a**) the corroded CeO_2 WE after electrochemical corrosion and (**b**) the pristine CeO_2 WE in the mass range of m/z^+ 1–355.

ToF-SIMS spectral results in **Figure S6** show no significant signals of fluorine containing fragments in the pristine and corroded CeO_2 WE. Therefore, the speculated fluorine contamination from Nafion is not a concern using this new approach. Known fluorine fragment peaks are the following, CF^+ m/z^+ 30.99, CF_2^+ m/z^+ 49.99, CF_3^+ m/z^+ 68.99, and $C_3F_3^+$ m/z^+ 92.99 [5].

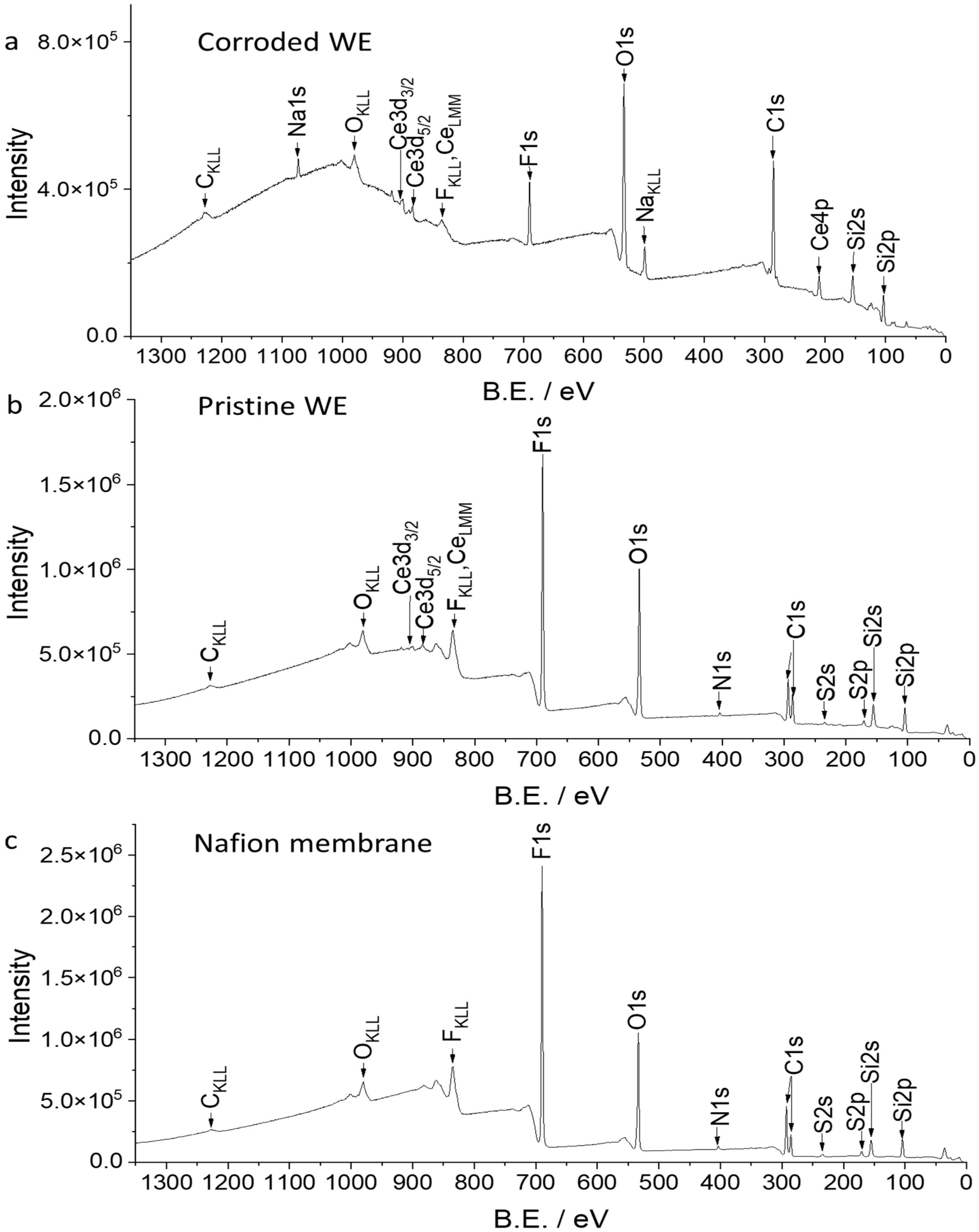

Figure S7. XPS wide scan spectral results of (**a**) the corroded CeO_2 WE after electrochemical corrosion, (**b**) the pristine CeO_2 WE, and (**c**) the Nafion membrane.

XPS wide scan spectra for the corroded, pristine, and Nafion control show a fluorine peak that is due to the Nafion membrane. Furthermore, substrate peaks (Si) can be observed in the corroded and pristine electrode, which indicates a thin film (< 5 nm) is present. The F(1s) peak is also observed to decrease in intensity, which may indicate some stripping of the Nafion membrane.

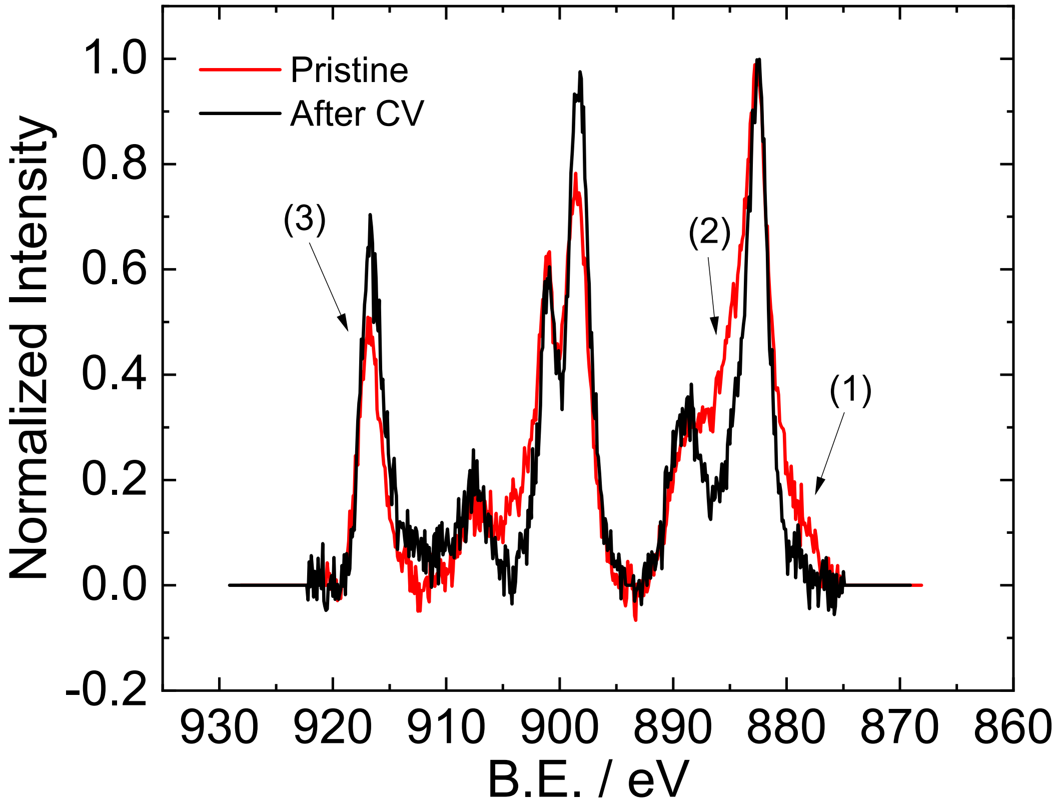

Figure S8. Normalized Ce(3d) spectra before and after cyclic voltammetry. Regions indicative of Ce^{3+} are marked (1), (2), and (3), respectively.

Before CV, features consistent with Ce^{3+} can be observed. (1) shows a shoulder at ~879 eV and (2) shows a shoulder at ~882eV, which indicates the 3+ oxidation state of ceria is present. Furthermore, the peak annotated as (3) (916.7 eV) is lower in intensity as compared to after CV, which further confirms the presence of Ce^{3+}.

Supplemental Tables

Table S1. XPS peak positions and corresponding FWHM values for the electrode analyzed before CV.

**Before CV**				
	Ce^{4+} $3d_{5/2}$	Ce^{4+} $3d_{5/2}$	Ce^{3+} $3d_{5/2}$	Ce^{3+} $3d_{5/2}$
Position (eV)	879.9	882.6	884.3	888.4
FWHM	2.5	2.5	2.4	3.5
Peak height ratio	1.6		0.5	

Two oxidation states of Ce (3+ and 4+) are observed and labeled according to the multiplet peak positions reported for Ce [6]. The FWHM maximum values are also reported. The normalized peak height was found by multiplying the height of the peak above the background by the FWHM and then taking the ratio, which agree with those reported by Paparazzo [7].

Table S2. XPS peak positions and corresponding FWHM values for the electrode analyzed after CV.

**After CV**		
	Ce^{4+} $3d_{5/2}$	Ce^{4+} $3d_{5/2}$
Position	882.7	888.8
FWHM	2.5	3.5
Peak height ratio	1.9	

After electrochemical cycling, one oxidation of Ce (4+) was observed with reported positions matching those reported in the literature. The peak height ratio was calculated using the same method as Paparazzo presented [7]. However, the peak height ratio is higher, which is likely due to the low signal-to-noise ratio.

References

1. Liu, B.; Yu, X.Y.; Zhu, Z.; Hua, X.; Yang, L.; Wang, Z. In situ chemical probing of the electrode-electrolyte interface by ToF-SIMS. *Lab Chip* **2014**, *14*, 855–859.
2. Yu, J.; Zhou, Y.; Hua, X.; Liu, S.; Zhu, Z.; Yu, X.Y. Capturing the transient species at the electrode-electrolyte interface by in situ dynamic molecular imaging. *Chem. Commun.* **2016**, *52*, 10952–10955.
3. Yang, L.; Yu, X.Y.; Zhu, Z.; Iedema, M.J.; Cowin, J.P. Probing liquid surfaces under vacuum using SEM and ToF-SIMS. *Lab Chip* **2011**, *11*, 2481–2484.
4. Guo, R.; Talma, A.G.; Datta, R.N.; Dierkes, W.K.; Noordermeer, J.W.M. Novel Surface Modification of Sulfur by Plasma Polymerization and its Application in Dissimilar Rubber-Rubber Blends. *Plasma Chem. Plasma Process.* **2010**, *30*, 679–695.
5. Ferrari, S.; Ratner, B.D. ToF-SIMS quantification of albumin adsorbed on plasma-deposited fluoropolymers by partial least-squares regression. *Surf. Interface Anal.* **2000**, *29*, 837–844.
6. Zhu, Y.; Jain, N.; Hudait, M.K.; Maurya, D.; Varghese, R.; Priya, S. X-ray photoelectron spectroscopy analysis and band offset determination of CeO_2 deposited on epitaxial (100), (110), and (111)Ge. *J. Vac. Sci. Technol. B* **2014**, *32*, 011217.
7. Paparazzo, E. Use and mis-use of X-ray photoemission spectroscopy Ce3d spectra of Ce_2O_3 and CeO_2. *J. Phys. Condens. Matter* **2018**, *30*, 343003.
